# A Case of Neuralgia of the Face Cured by Exsection of Part of the Nerve

**Published:** 1855-10

**Authors:** Wm. P. Casseday

**Affiliations:** Louisville, Prosector and Clinical Assistant of the Same; New York


### ART. II.—A CASE OF NEURALGIA OF THE FACE CURED BY EXSECTION OF PART OF THE NERVE. BY PROF. J. M. CARNOCHAN, OF THE NEW YORK MEDICAL COLLEGE.

REPORTED BY WM. P. CASSEDAY, M. D., OF LOUISVILLE, PROSECTOR AND CLINICAL ASSISTANT OF THE SAME.

THE following case of Neuralgia is one of great interest in reference to the cure of that disease by operation. As will be seen it was a case of a very stubborn nature, and it seemed almost useless to operate. This, however, seemed the only alternative, notwithstanding the seven different operations which had been performed. The same nerve had been divided twice by Dr. Jno. Watson, once by Dr. Willard Parker, three times by Dr. Mott, and once by Dr. Detmold.

The patient gave the following history of his case: Named J. C. Forbes; aged forty-seven, citizen of Hoboken, N. J.; married; of a nervo-bilious temperament; occupation, house carpenter.

He first noticed, in May, 1849, that when he drew his handkerchief across his upper lip and the end of his nose, a sharp, lancinating pain would shoot from near the middle of the lip, along the fossa at the junction of the nose and cheek on the left side, up to the inner angle of the eye. Also that when he touched his lip with his tongue, or attempted to swallow, he felt the same pain. These symptoms continued until autumn of the same year, without any change or any additional symptoms, when they wholly disappeared, and did not return until the next spring. In the spring of 1850 they returned in the same way, slight at first, but they increased much, and the patient thinking the disease might be caused by some bad teeth, and partly by the advice of a dentist, had every tooth extracted, except a small stump, on the right side of the lower jaw, which was overlooked. This gave no relief whatever. The disease increased and he suffered greatly with paroxysms over the whole left cheek. His sufferings were almost intolerable.

From this time until February 1852 the patient was under medical treatment, everything likely to do any good being tried, but in vain. In February, 1852, Dr. J. Watson dissected the entire cheek, from the superior maxilla, the dissection extending from the nose to the tuberosity of maxilla, and the malar bone, down to the alveolar border, and up to the orbit. It was made from the mouth with no external incision. This gave much relief until the next November when the paroxysms returned.

After this time until December he was treated occasionally with quinine. During the latter month he was again operated upon by the same surgeon, who made a V incision over the infra-orbital foramen, and divided the nerve coming out there. This again relieved him greatly, until autumn 1853, when the disease again appeared, and he was again put under medical treatment, but with no relief. Galvanism was tried at this time but ineffectually.

In January, 1854, Dr. Willard Parker, operated, making the same incisions as the last, and relieving the disease until August of the same year. The paroxysms then reappeared and continued until September, 1854, when Dr. Detmold operated, but with no good effect.

In October Dr. Mott divided the nerve by subcutaneous incision, and did the same in November. These operations afforded very slight relief, and on the 8th of January, 1855, Dr. Mott made a V incision, at the same spot at which Dr. Parker had made it, but more extended, and again divided the nerve. This afforded only partial relief. However the patient could attend to his business with little inconvenience.

In June, 1853, the disease again increased, and the patient went to the New York Hospital, and was under treatment there until August, when he applied to Dr. Carnochan, who treated him for a short time with Donovan's solution, aconite internally and externally, and an ointment of the extracts of belladonna, hyoscyamus and strammonium, but with no effect.

August 29th, the patient was no better, and it was decided to perform another operation.

August 31st, the patient thought he was a little better, but the pain still continued. He had been using veratrine since the last date.

This being the day appointed for the operation the patient was put under the influence of chloroform, when a V incision was made over the foramen, its base being turned toward the orbit, and the flap thus made dissected from the bone, and the dissection extended into the orbit, on its floor, for one inch. Proceeding cautiously, partly with the knife and partly tearing the tissues the foramen was found. It was difficult to isolate the nerve on account of the extensive hard cicatrix, the result of seven different operations at the same point. But the foramen being found and the nerve isolated, a small piece of the bone was removed from the edge of the orbit, and a part of its floor with a chisel and hammer. The canal was thus laid open half an inch, and a piece of the nerve in its entire diameter, was excised, being all that part exposed by the removal of the roof of the canal.

The operation was excessively painful as the patient could not be easily kept under the influence of the anæsthetic for any length of time. There seemed to be a peculiarly strong resistance to the operation of the chloroform. Although he was apparently insensible, when the nerve was touched he would start and in a very few seconds be perfectly sensible and of course in great pain. Five ounces of chloroform were used during the operation.

Almost immediately after the excision he drank a glass of brandy and water with perfect ease, which for years before this was impossible, on account of the intolerable pain caused by it. He was sent to bed perfectly relieved of pain, and an anodyne administered.

September 1st, I saw the patient; I found him sitting up, in fine spirits, suffering no pain; he was awake much of the night before, but that was from excitement, not pain. He could eat without the least inconvenience, except that arising from the loss of his teeth.

September 5th, the patient presented himself at the office. He had no pain nor any constitutional symptoms. The dressings were removed and the surface of the wound was found to be freely suppurating. He could still eat without pain and was in as good condition as could be desired under the circumstances.

September 5th, was still in good condition. He complained of some soreness in the wound, which was covered with healthy granulations. His digestion was not good, furred tongue, etc.; he was ordered.

℞ Iodide Potass. ʒjss.

Tinct. Conium ʒij.

Water ℥viii. M. S.

Tablespoonful three times a day.

From this time the case progressed favorably. The wound is now entirely healed and the patient at work, having had no return of his disease. He complained at one time of pain along the track of the portio-dura, where it makes its exit from the stylomastoid foramen, which can be accounted for by the small recurrent filament which joins the portio-dura from the ganglion of Meckel.

He had an attack of intermittent fever Sept. 11th and 12th, and again about the 17th to 20th.

It is to be remembered that although the operations performed before this by Prof. Carnochan, relieved the patient, yet the relief was at no time so complete and perfect. The patient says he was always in a worse condition immediately after those operations, but that after a certain time he recovered, and was much though not entirely relieved. But after this last the relief was immediate and entire. This seems to show that the better plan was to excise a portion of the nerve entirely rather than merely to divide it.

The case will be watched, and if there is any return of the disease it will be immediately reported.

New York, Oct. 1st, 1855.

